# Dr. Raymond A. Clifford

**Published:** 1918-05

**Authors:** 


**Dr. Raymond A. Clifford,** University of Michigan, Homeopathic Medical School, 1898, Jefferson Medical College, 1899, died in the Clifton Springs Sanitarium March 2, age 44. His home was in Ypsilanti, Mich.

